# COVID-19: ICU delirium management during SARS-CoV-2 pandemic—pharmacological considerations

**DOI:** 10.1186/s13054-020-03072-5

**Published:** 2020-06-23

**Authors:** Lauren J. Andrews, Scott T. Benken

**Affiliations:** Department of Pharmacy Practice, University of Illinois at Chicago College of Pharmacy, 833 South Wood Street, RM 164 MC 886, Chicago, IL 60612-7230 USA

The recent article by Kotfis and colleagues provides great insight regarding newly identified COVID-related risk factors contributing to ICU delirium, while also presenting valid potential solutions to barriers surrounding the implementation of the ABCDEF safety bundle for ICU liberation [1]. However, we feel that the omission of pertinent medication availability and selection limitations currently afflicting healthcare teams around the world warrants further discussion.

In the setting of medication shortages and anecdotally high sedation requirements in COVID patients, it has become increasingly necessary to utilize various combinations of second-line and adjunct therapy options in order to maintain appropriate levels of sedation, with indications for neuromuscular blockade and prone positioning further exacerbating this need [1]. Keeping the known sedation limitations of dexmedetomidine in mind [2], clinicians may be more inclined to use benzodiazepine infusions if unable to obtain adequate supplies of common first-line agents (e.g., fentanyl, propofol). Despite the well-documented association between benzodiazepines and delirium, this approach may be unavoidable for a large number of institutions. Ketamine may be a possible solution, however, as more data has recently emerged regarding its safety and efficacy as an adjunct therapy for analgosedation [3].

In recognition of this change in practice, it is imperative for healthcare teams to thoroughly evaluate the benefits and risks of adjunct agents initiated for the purpose of minimizing intravenous sedative requirements. While the strategy of using intermittent antipsychotics to prevent suppression of respiratory drive and subsequently aid in the promotion of daily spontaneous breathing and awakening trials could be considered, there are important considerations regarding adverse effects associated with antipsychotics that are likely to be exacerbated in COVID patients. Early recommendations for hydroxychloroquine therapy, empiric community-acquired pneumonia coverage with azithromycin for atypical bacterial co-infections, and the possibility of COVID-related cardiomyopathies all increase the risk of cardiac decompensation [4], with the addition of antipsychotics only serving to amplify the risk of QTc prolongation and worsen morbidity. In an attempt to avoid the preventable adverse events associated with antipsychotics, a recommendation endorsing agents like ketamine, valproic acid, or clonidine may be more appropriate options when implementing intravenous sedative-sparing strategies [3, 5].

With more information discussing the pharmacological limitations evidenced during the COVID era, readers may be better prepared to address the COVID-related risk factors for delirium when factoring in the potential adverse outcomes associated with second-line agents like benzodiazepines and antipsychotics during medication shortages.

## Authors’ response

Katarzyna Kotfis, Shawniqua Williams Roberson, Jo Ellen Wilson, Wojciech Dabrowski, Brenda T. Pun, and E. Wesley Ely

Dear Editor,

We appreciate the thoughts and questions posed by Dr. Andrews and Dr. Benken about pharmacological considerations related to our manuscript entitled, “COVID-19: ICU delirium management during SARS-CoV-2 pandemic.” We agree wholeheartedly that resource constraints and drug shortages posed by the pandemic mandate that we develop alternative means by which to keep patients safe and comfortable while they are suffering on life support from severe acute respiratory distress syndrome (ARDS), shock, and other types of organ dysfunction. The rise in benzodiazepine use was especially disheartening, as we know that this creates a very high likelihood of our patients spending more time being delirious. This, in turn, increased the risk of acquired dementia and other elements of post-intensive care syndrome (PICS) resulting from immobilization that commonly occurs when someone is profoundly delirious.

The authors suggest that ketamine, valproic acid, and clonidine are more appropriate options than antipsychotics. In some circumstances, that may very well be true, and we are not inclined to disagree outright other than to say that there is a paucity of data upon which to base such a decision. In fact, we know of not a single randomized controlled trial that would support such a decision. Anecdote and expert opinion do serve a place in difficult times, and the COVID-19 pandemic has most certainly in some hospitals and scenarios created desperation. We believe, however, that such deviations from data should be the exception rather than the rule. In this case given by Dr. Andrews and Dr. Benken, the double use of hydroxychloroquine and azithromycin itself must be questioned as unsupported by data, and thus, first why increase the likelihood of QTc prolongation by these two anecdotally chosen agents. Perhaps instead they should be removed from the patient’s regimen unless they are part of an ongoing clinical trial or some convincing evidence for their benefit becomes available. At the time of this writing, there is not enough data to support this decision. Though it is now known that antipsychotics do not treat delirium itself [6–9], it is the case that they did not increase the risk of torsades de pointes or have other notable safety issues in the clinical trials recently published.

## Data Availability

Not applicable
